# The Potential of Secondary Metabolites from Plants as Drugs or Leads against Protozoan Neglected Diseases—Part III: In-Silico Molecular Docking Investigations

**DOI:** 10.3390/molecules21101389

**Published:** 2016-10-19

**Authors:** Ifedayo Victor Ogungbe, William N. Setzer

**Affiliations:** 1 Department of Chemistry and Biochemistry, Jackson State University, Jackson, MS 39217, USA; ifedayo.v.ogungbe@jsums.edu; 2 Department of Chemistry, University of Alabama in Huntsville, Huntsville, AL 35899, USA

**Keywords:** *Leishmania*, *Trypanosoma*, *Plasmodium*, natural products drug discovery

## Abstract

Malaria, leishmaniasis, Chagas disease, and human African trypanosomiasis continue to cause considerable suffering and death in developing countries. Current treatment options for these parasitic protozoal diseases generally have severe side effects, may be ineffective or unavailable, and resistance is emerging. There is a constant need to discover new chemotherapeutic agents for these parasitic infections, and natural products continue to serve as a potential source. This review presents molecular docking studies of potential phytochemicals that target key protein targets in *Leishmania* spp., *Trypanosoma* spp., and *Plasmodium* spp.

## 1. Introduction

Parasitic protozoal diseases continue to be a cause of considerable morbidity and mortality, particularly in underdeveloped countries around the world. These diseases include malaria [1], Chagas disease [2], human African trypanosomiasis [3], and leishmaniasis [4]. Current chemotherapeutic options for these neglected diseases can have severe side effects, may be ineffective, or even non-existent, and in cases where drug treatment is available, resistance is emerging [5,6,7,8]. Thus, there is a need to discover and develop of new chemotherapeutic agents for these parasitic infections. Natural products have been served as important leads for drug development and databases of natural products provide a convenient source for virtual screening against drug targets [9], including parasitic protozoal diseases [10,11,12]. Natural products offer important complementary opportunities in drug discovery: (a) They occupy different regions of biologically relevant chemical space [13], including abundant oxygen-containing functionalities (rarely nitrogen) and high degrees of chirality and complexity [14]; (b) although outside the “rule-of-five”, numerous natural products have proven to be efficacious drugs [15]; (c) they have been optimized for activity, including active transport, by evolution [16]; and (d) natural products serve as lead structures for semisynthetic modification to improve activity, selectivity, or bioavailability [17]. In this review, we present in-silico efforts at natural product drug discovery for neglected parasitic protozoal diseases; molecular docking of phytochemical ligands with potential parasitic protein targets (Table 1).

## 2. Parasite Molecular Targets

Numerous protozoal proteins have been identified as druggable or potentially drugable targets, including deoxyuridine triphosphate nucleotidohydrolase, dihydroorotate dehydrogenase, farnesyl diphosphate synthase, glyceraldehyde 3-phosphate dehydrogenase, nucleoside diphosphate kinase B, pteridine reductase, pyruvate kinase, sterol 14α-demethylase, triosephosphate isomerase, and trypanothione reductase [10], and many of these have been characterized crystallographically (see Table 1). These protein crystal structures serve as—structural models for in-silico screening using molecular docking techniques. Often, there are different structures, usually with different co-crystallized ligands, that can provide slightly different and complementary binding sites for docking studies.

## 3. Molecular Docking Studies

Molecular docking has become one of the most important modeling tools in modern drug discovery. It is a very convenient and cheap means to study protein-ligand interactions. It can be used to rank compounds for prioritization in lead discovery and development. It can also be used to identify potential inhibitors, substrates, activators or binding partners from compound libraries that contains few hundreds to millions of compounds. Several recent reviews on molecular docking have appeared [357,358,359,360], so molecular docking principles and approaches will not be covered here.

Molecular docking has become a well-accepted complement to X-ray crystallography and NMR spectroscopy in studying drug—drug target interactions. It also gives the medicinal chemist a means to access certain ligand binding poses that even X-ray crystallography and NMR spectroscopy may not inform the most experienced structural biologist thereby aiding the medicinal chemist in the creative enterprise of structure-based drug design. In some cases, it has become a replacement or a complement to high throughput compound screening.

Despite current and potential applications, as well as the successes of molecular docking in drug discovery, there remain limitations and caveat in the interpretation of results from molecular docking studies. These limitations stem, mostly, from the inability of the scoring functions in molecular docking algorithms to account for local and global macromolecular dynamics, in addition to inability to accurately predict covalent interactions and solvent accessibilities: (1) in most cases the protein is modeled as a rigid structure without flexibility; (2) solvation of the active/binding site and of the ligand is usually excluded; (3) free-energy estimation of protein-ligand complexes is largely ignored [357,361,362]. Molecular docking methodology, cavity definition and search algorithms, and thermodynamic scoring functions continue to improve, however [363].

Molecular docking has increasing found use in drug discovery programs focused on tropical diseases. The applications include target-based screening of natural products libraries or databases [364,365]. Popular natural product databases include the *Dictionary of Natural Products* [366], Napralert [367], and the ZINC natural products database [368].

Table 2 lists popular molecular docking software recently used for virtual screening of natural product libraries. The list is not meant as an endorsement, but does reflect the current availability of molecular docking software. In addition, several other commercial and freeware molecular docking packages are available. There are, however, additional effects to be considered in docking studies with natural products: (1) many natural products may have poor bioavailability due to limited solubility, membrane permeability, hydrolysis, or other metabolic transformations; (2) the ligands may also target homologous isozymes in humans and cause serious side effects; (3) the docking studies do not account for possible synergism with the bioactive antiparasitic compounds.

In spite of the above limitations, molecular docking studies of phytochemical ligands with identified protein targets provide the possibilities to identify natural compounds that may themselves function as efficacious drugs, may serve as lead structures for chemical modification and optimization, or provide structural templates for de novo drug synthesis.

Some published reports have focused on natural products that are biologically active against one or more protozoan organism or any of their validated drug targets [374,375,376,377,378] while other works have focused on natural products or phytochemicals that were isolated from plants with historical ethnomedicinal therapeutic use [379,380]. Molecular docking has been used to identify, in silico, the selectivity of some compounds or classes of compounds for specific protozoan drug targets. In the reports by us about the selectivity of antiparasitic isoprenoid derivatives for drug targets from *Leishmania* spp., for example, antiparasitic monoterpenoids were found to selectively dock to *L. infantum* nicotinamidase, *L. major* uridine diphosphate-glucose pyrophosphorylase, and methionyl t-RNA synthetase, while germacranolide sesquiterpenoids were selective for *L. major* methionyl t-RNA synthetase, and dihydroorotate dehydrogenase [375]. It was also shown in that work that diterpenoids generally favored docking to *L. mexicana* glycerol-3-phosphate dehydrogenase. In addition, the tetranortriterpene limonoids showed some selectivity for *L. mexicana* glycerol-3-phosphate dehydrogenase and *L. major* dihydroorotate dehydrogenase while withanolides docked more selectively with *L. major* uridine diphosphate-glucose pyrophosphorylase.

**Figure 1:**
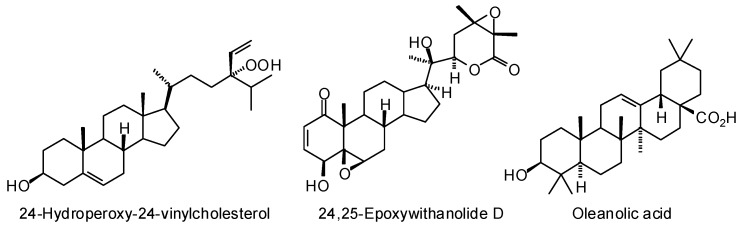
Phytochemical ligands with encouraging docking properties with *L. infantum* sterol 14α-demethylase (LinfCYP51).

**Figure 2:**
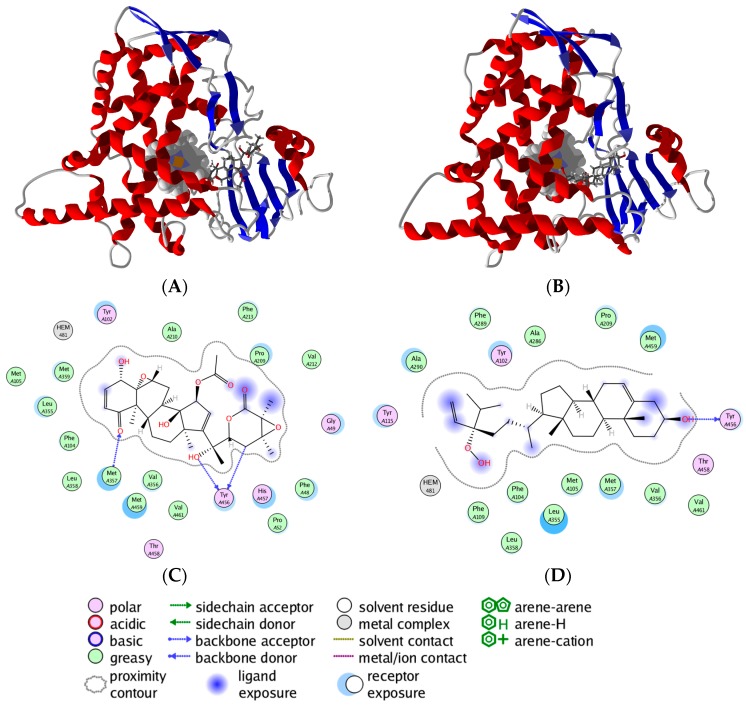
The lowest energy docking poses of *L. infantum* sterol 14α-demethylase (LinfCYP51) with 24,25-epoxywithanolide D (**A**) and 24-hydroperoxy-24,25-vinylcholesterol (**B**) (stick figures); The heme cofactor is shown as a space-filling model. LinfCYP51 was predicted to have hydrogen bonding interactions with 24,25-epoxywithanolide D through the backbones of Tyr 458 and Met 357 residues, in addition, to van der Waals interactions with Leu 355, Met 359 and Val 356 (**C**); In the case of 24-hydroperoxy-24,25-vinylcholesterol, hydrogen bonding with Tyr 456 was predicted as well (**D**). Extensive van der Waals interactions between the hydroperoxy sterol and Met 357, Met 459 and Phe 104 of LinfCYP51 were also predicted.

Also, although not surprising, were the strong docking preference of several steroids and triterpenoids for *L. infantum* sterol 14α-demethylase (LinfCYP51). Of particular note is the strong docking preference of the hydroperoxy sterol 24-hydroperoxy-24, 25-vinylcholesterol and 24,25-epoxywithanolide D (Figure 1) to LinfCYP51 (Figure 2). In vitro evaluation of these compounds as possible inhibitors of LinfCYP51 remains to be tested, but in vitro antileishmanial screening and in silico docking with LinfCYP51 of oleanolic acid (Figure 1) corroborate these findings [381].

### 3.1. Leishmania and Trypanosoma Targets

**Figure 3:**
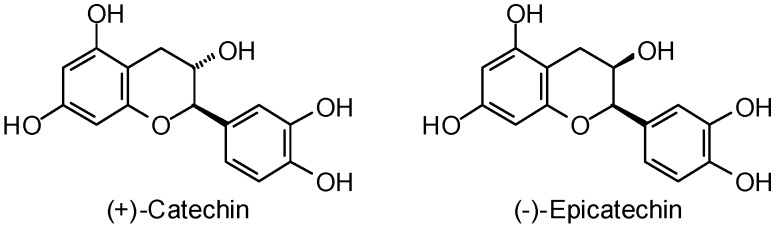
*Leishmania amazonensis* arginase inhibitors.

The flavonoids (+)-catechin and (−)-epicatechin (Figure 3) have been shown to be effective inhibitors of *Leishmania amazonensis* arginase with IC_50_ values of 0.77 and 1.8 μM, respectively [382]. Molecular docking (MolDock) of these compounds has revealed differences in their interactions with the amino acid residues of the active site of arginase. (+)-Catechin docks in the active site with primary hydrogen-bonding interactions to Ala192, Thr257, and Asp141. In contrast, the primary hydrogen-bonding contacts for (−)-epicatechin were with Ser150, His154, Asp245, Asn 152, Thr257, and Asn243.

**Figure 4:**
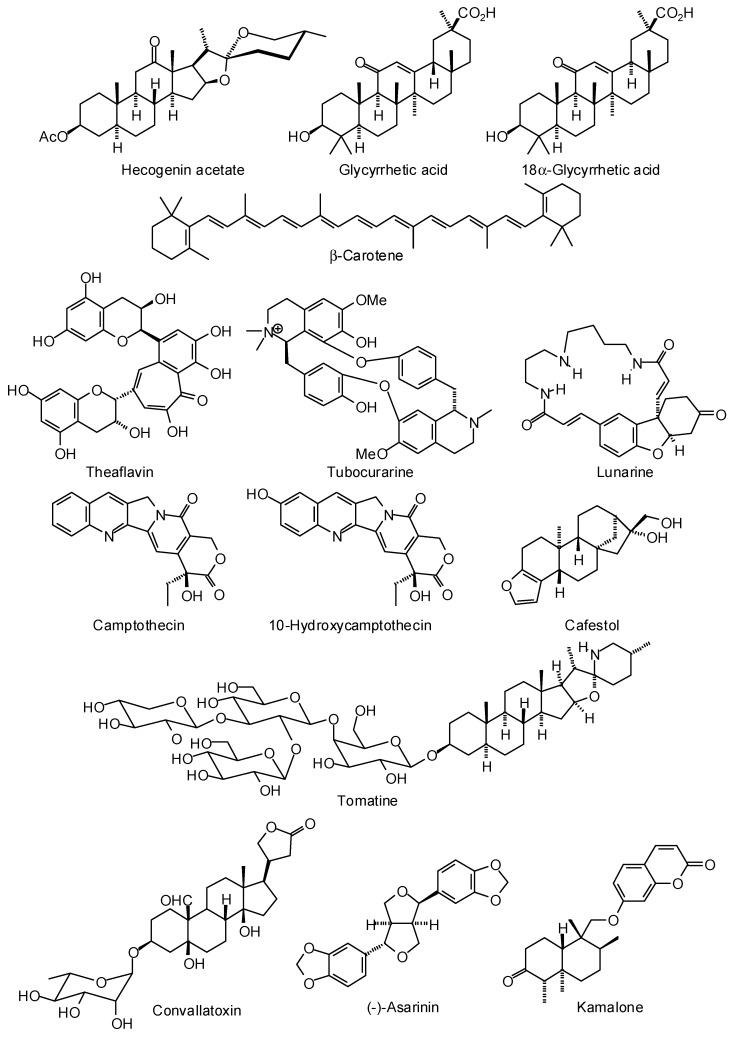

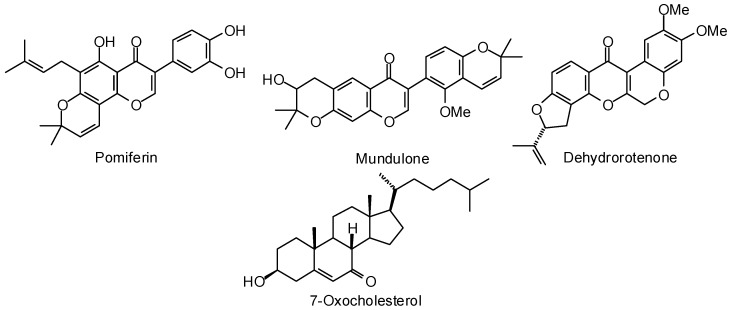
Phytochemical ligands with encouraging docking properties with *Leishmania* trypanothione synthase.

Venkatesan and co-workers [383] carried out a docking investigation of *Leishmania* trypanothione synthase with the MS Discovery database of 800 compounds, using AutoDock. The best phytochemicals docking to the crystal structure of *L. major* trypanothione synthase were theaflavin, hecogenin acetate, β-carotene, glycyrrhetic acid, 18α-glycyrrhetic acid, convallatoxin, tubocurarine, and lunarine (Figure 4). Strongly docking phytochemical ligands to a homology-modeled structure of *L. donovani* trypanothione synthase included 10-hydroxycamptothecin, camptothecin, tubocurarine, tomatine, cafestol, (−)-asarinin, pomiferin, 7-oxocholesterol, mundulone, and dehydrorotenone (Figure 4). In a complementary examination of antileishmanial sesquiterpenoids, Bernal and Coy-Barrera found the coumarin-derived sesquiterpenoid kamalone to be a strongly docking ligand for *L. major* trypanothione synthase [378].

**Figure 5:**
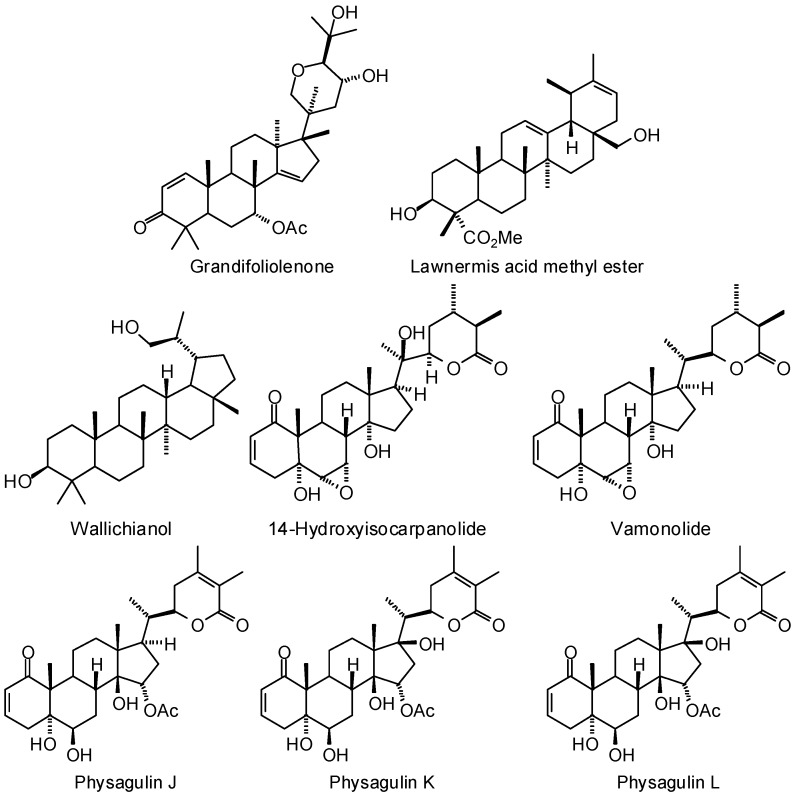

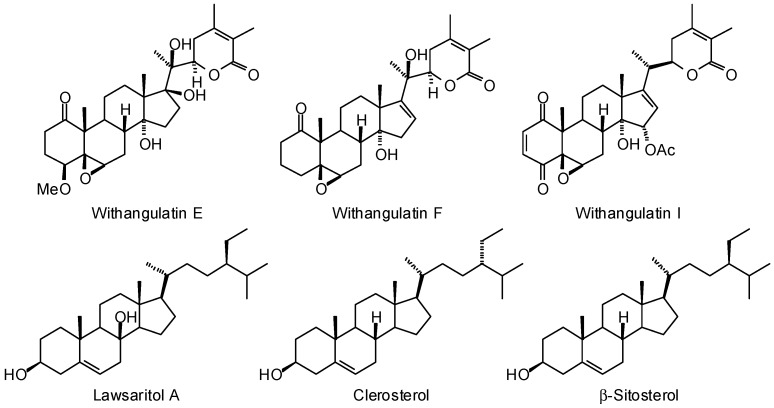
Phytochemical ligands that docked selectively with *Trypanosoma brucei* 14α-demethylase.

An in-silico screening study (MolDock) of antiparasitic medicinal plants from West Africa has revealed several phytochemicals with strong, selective docking to a number of *Trypanosoma brucei* protein targets [379]. This investigation revealed that several triterpenoid and steroid ligands (e.g., grandifoliolenone, lawnermis acid methyl ester, lawsaritol A, wallichianol, 14-hydroxy-isocarpanolide, physagulins J, K, and L, vamonolide, withangulatins E, F, and I, clerosterol, and β-sitosterol, Figure 5) were selective for *T. brucei* sterol 14α-demethylase.

**Figure 6:**
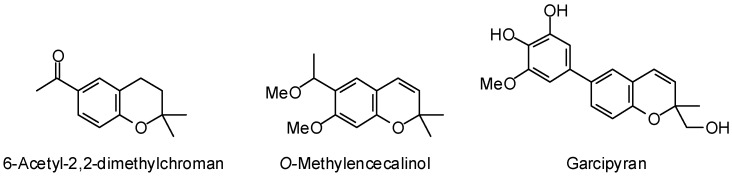
Phytochemical ligands that docked selectively with *Trypanosoma brucei* triosephosphate isomerase.

**Figure 7:**
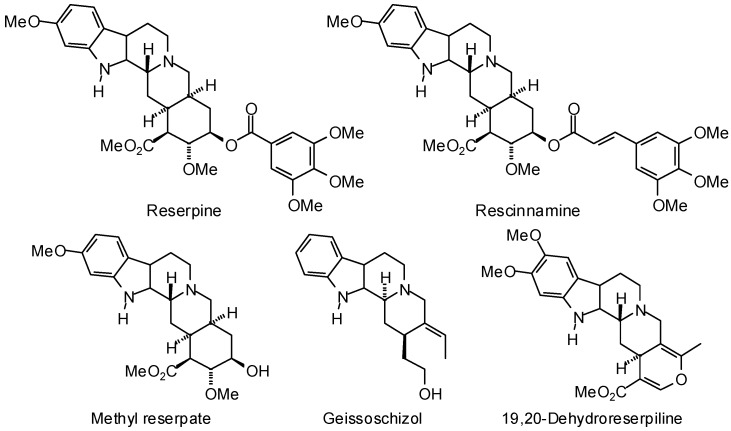
Phytochemical ligands with encouraging docking properties with *Trypanosoma brucei* UDP-galactose-4′-epimerase.

Chromenes (e.g., 6-acetyl-2,2-dimethylchroman, *O*-methylencecalinol, and garcipyran, Figure 6), showed preferential docking to *T. brucei* triosephosphate isomerase, while indole alkaloids (e.g., reserpine, rescinnamine, methyl reserpate, geissoschizol, and 19,20-dehydroreserpiline, Figure 7) exhibited notably low docking energies for *T. brucei* UDP-galactose-4′-epimerase.

**Figure 8:**
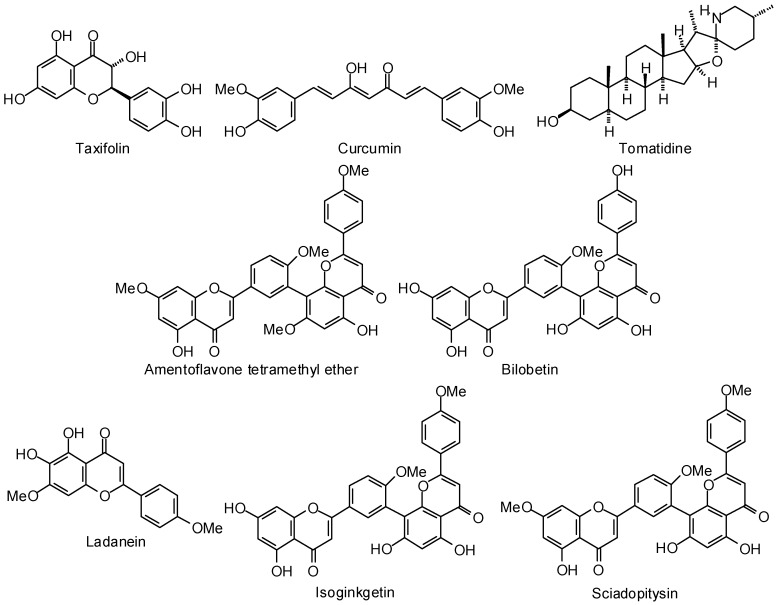

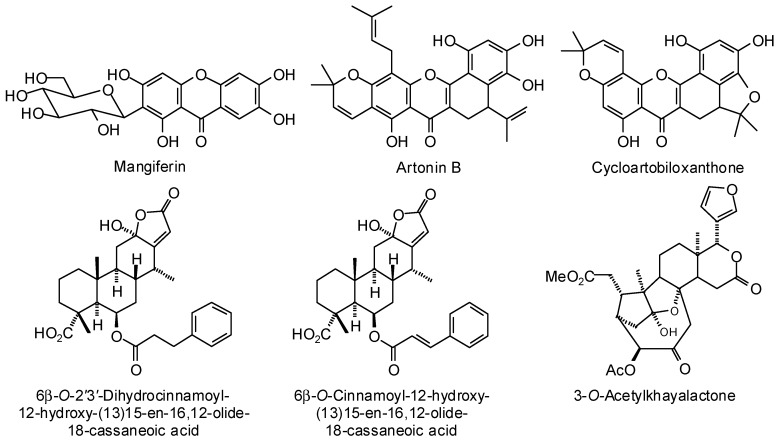
Phytochemical ligands with encouraging docking properties with *Leishmania* trypanothione reductase.

Trypanothione reductase has been investigated as a protein target for several parasitic protozoa, including *Leishmania* spp., *T. brucei*, and *T. cruzi* [384]. The flavonoid taxifolin (Figure 8) was found to dock (AutoDock) at the active site of *L. infantum* trypanothione reductase [385]. Similarly, Ribeiro and co-workers, using MolDock, found the flavonoid ladanein to dock strongly with *L. infantum* trypanothione reductase [386]. Ogungbe and co-workers [377] have found that dimeric flavonoids such as amentoflavone tetramethyl ether, bilobetin, isoginkgetin, and sciadopitysin (Figure 8), dock (MolDock) much more strongly to *L. infantum* trypanothione reductase than monomeric flavonoids, although these dimeric compounds generally violate Lipinski’s rule of five [387]. The glycosylxanthone mangiferin was shown to dock (AutoDock) in the active site of *L. infantum* trypanothione reductase [388]. Prenylated xanthones have also demonstrated notably strong docking (MolDock) to *L. infantum* trypanothione reductase [377]. Of the polyphenolic ligands examined, Ogungbe and co-workers [377] found the flavonoids artonin B and cycloartobiloxanthone (Figure 8) to show selective docking to this protein target. Strongly docking (MolDock) terpenoid ligands for *L. infantum* trypanothione reductase included the cassane diterpenoids 6β-*O*-2′3*′*-dihydrocinnamoyl-12-hydroxy-(13)15-en-16,12-olide-18-cassaneoic acid and 6β-*O*-cinnamoyl-12-hydroxy-(13)15-en-16,12-olide-18-cassaneoic acid, and the limonoid 3-*O*-acetylkhayalactone (Figure 8) [375]. As part of her M.S. thesis, Ritika Chauhan developed a homology model of *L. donovani* trypanothione reductase and found curcumin (Figure 8) and curcumin derivatives to be strongly docking using AutoDock [389]. The alkaloid tomatidine (Figure 8), the aglycone of tomatine, from *Solanum* spp. was identified as a potential inhibitor of *Leishmania infantum* trypanothione reductase using molecular docking (AutoDock) by Venkatesan and Dubey [390]. Tomatidine is a known inhibitor of multidrug resistance transporter in human cancer cells [391] and a more recent report has indicated that tomatidine affects sterol biosynthesis in promastigotes of *Leishmania amazonensis* and can lead to mitochondrial dysfunction in those parasites [392]. There is no known published report on the inhibitory activity of the compound on trypanothione reductase, but the authors suggested that tomatidine has a structural scaffold that makes it a potential inhibitor of trypanothione reductase.

**Figure 9:**
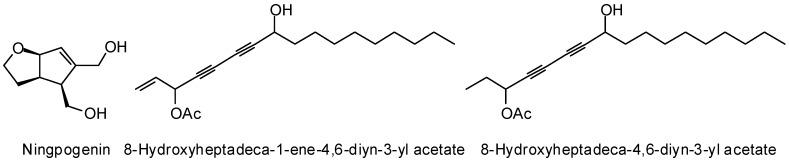

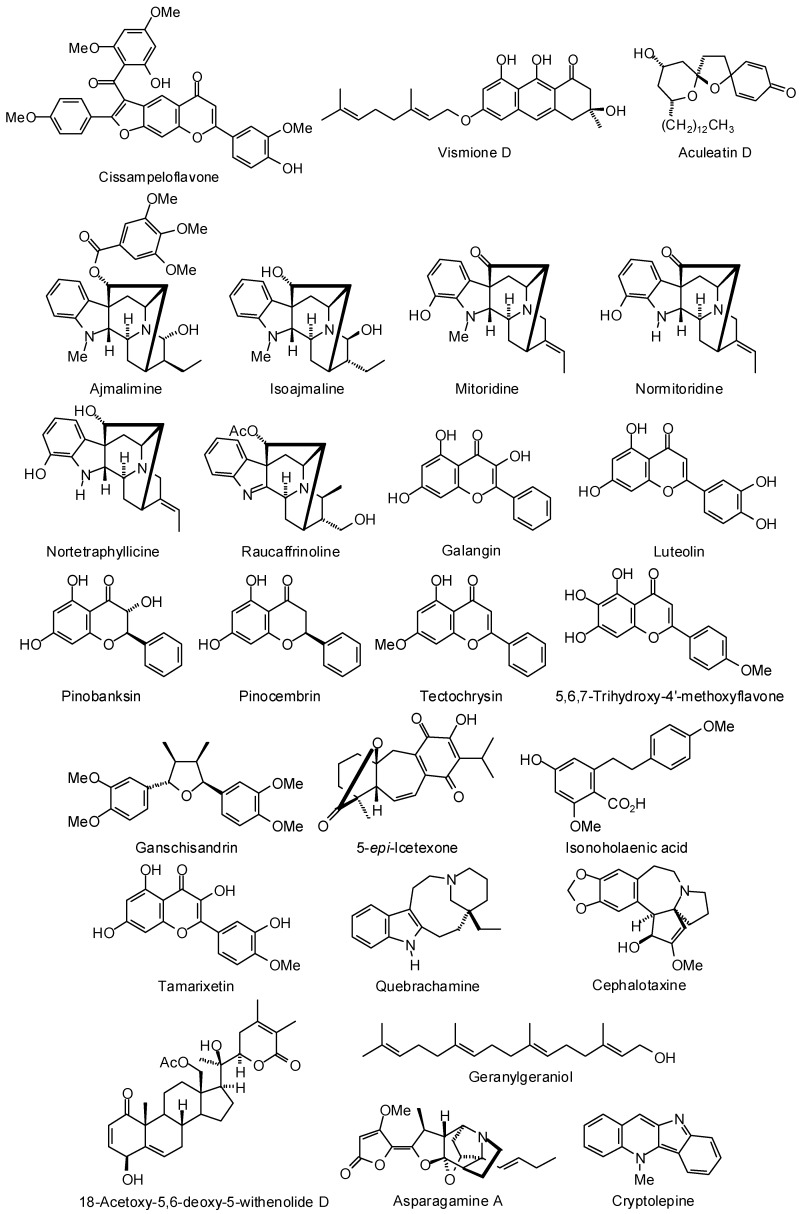

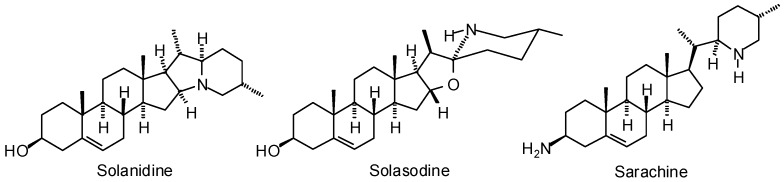
Phytochemical ligands with encouraging docking properties with *Trypanosoma* trypanothione reductase.

Several antitrypanosomal phytochemicals have shown strong docking (MolDock) to *T. brucei* trypanothione reductase, including the iridoid ningpogenin, the diacetylenes 8-hydroxyheptadeca-1-ene-4,6-diyn-3-yl acetate and 8-hydroxyheptadeca-4,6-diyn-3-yl acetate, the flavonoid cissampeloflavone, the anthracenone vismione D, and aculeatin D (Figure 9) [393]. A number of *Rauwolfia vomitoria* indole alkaloids (ajmalimine, isoajmaline, mitoridine, normitoridine, nortetraphyllicine, and raucaffrinoline, Figure 9) have shown selective docking (MolDock) to this protein target [379]. Numerous phytochemicals have shown antiparasitic activity against *T. cruzi* [394] and a molecular docking study (MolDock) has revealed several of these to show selective docking to *T. cruzi* trypanothione reductase [380]. A number of flavonoids (galangin, luteolin, pinobanksin, pinocembrin, tectochrysin, and 5,6,7-trihydroxy-4′-methoxyflavone, Figure 9), as well as the lignan ganschisandrin, the diterpenoids 5-*epi-*icetexone, and the stilbenoid isonohalaenic acid (Figure 9), showed notable selective docking to *T. cruzi* trypanothione reductase. Additionally, several flexible, hydrophobic ligands, geranylgeraniol (Figure 9) and C_17_ fatty alcohol derivatives from *Persea americana*, also docked strongly. In a similar study, the flavonoid tamarixetin showed strong selective docking to *T. cruzi* trypanothione reductase [386]. In a molecular docking search of alkaloids, Argüelles and co-workers concluded that quebrachamine, cephalotaxine, cryptolepine, tomatidine (Figure 8), solanidine, and solasodine (Figure 9) could serve as lead molecular scaffolds for *T. cruzi* trypanothione reductase inhibitors [395]. Likewise, the alkaloid asparagamine A docked selectively to both *L. infantum* and *T. cruzi* trypanothione reductase [396]. An AutoDock study by Saha and Sharma revealed the withanolide 18-acetoxy-5,6-deoxy-5-withenolide D and the steroidal alkaloid sarachine (Figure 9) to be strongly docking phytochemical ligands for *T. cruzi* trypanothione reductase [397].

**Figure 10:**
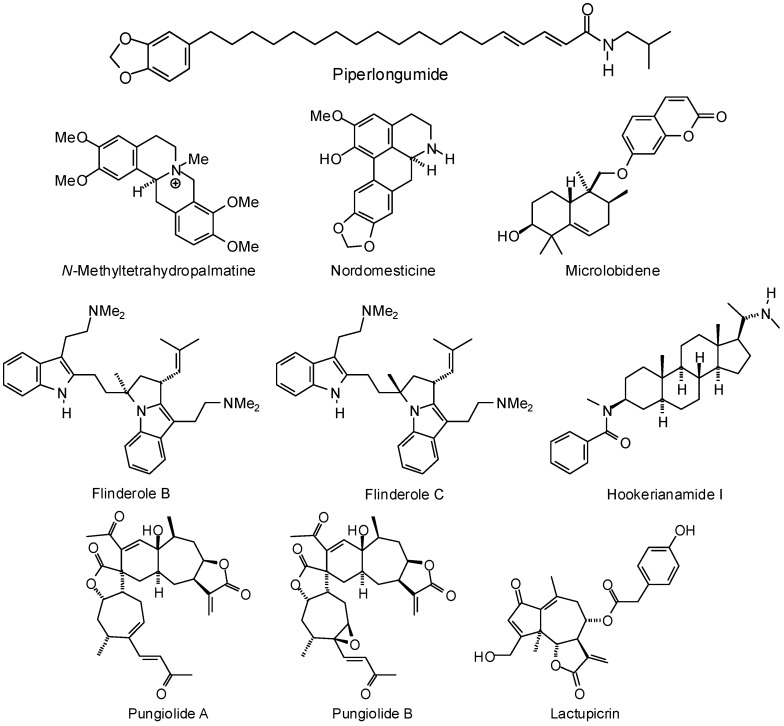
Phytochemical ligands with encouraging docking properties with parasite pteridine reductases.

Parasitic trypanosomatids salvage pterins from their host organisms using pteridine reductase (PTR1), and this enzyme is a potential target for antiparasitic drug development [242]. Sahi and co-workers have carried out in vitro antileishmanial screening of constituents from *Piper longum* fruit and found the alkaloid piperlongumide (Figure 10) to be the most active compound [398]. A molecular docking study using a homology model for *L. donovani* PTR1 has suggested that this protein may be the target for piperlongumide. An in-silico screening study of antitrypanosomal phytochemicals has found the alkaloids *N*-methyltetrahydropalmatine, nordomesticine (Figure 10), and sarachine (Figure 9) to dock preferentially to *T. cruzi* pteridine reductase [380]. The bis-indole alkaloids flinderole B and flinderole C, as well as the steroidal alkaloid hookerianamide I (Figure 10) showed docking preferences for *L. major* pteridine reductase 1 [374]. Bernal and Coy-Barrera have examined several antileishmanial sesquiterpenoids for docking (AutoDock Vina) to *L. major* PTR1 and found pungiolide A, pungiolide B, and microlobidene (Figure 10), to be the strongest docking ligands [378]. In another study, the guaianolide sesquiterpenoid lactupicrin (Figure 10) docked strongly to *L. major* PTR1 [375].

**Figure 11:**
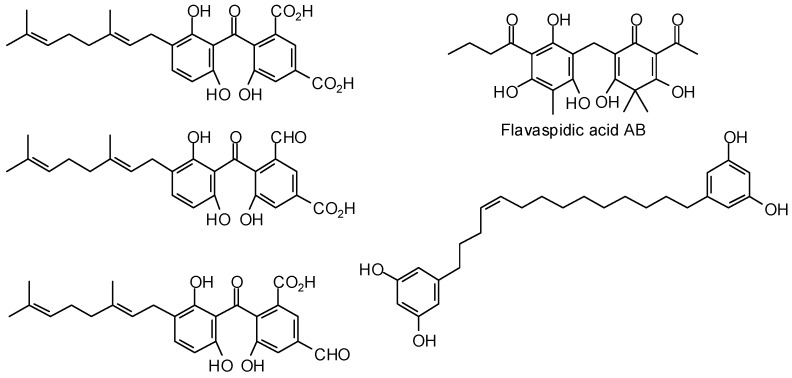
Phytochemical ligands with encouraging docking properties with *Trypanosoma brucei* glyceraldehyde 3-phosphate dehydrogenase.

Herrmann and co-workers have carried out an in-silico screening of a natural products library of 700 structures against *T. brucei* glyceraldehyde-3-phosphate dehydrogenase (TbGAPDH) [399]. These investigators were able to identify 13 “hits” based on the molecular docking and of these, five compounds (three geranylated benzophenones, flavaspidic acid AB, and a bis-resorcinyl tetradecene derivative, Figure 11) showed significant in vitro inhibitory activity against recombinant TbGAPDH as well as *T. brucei rhodesiense*.

An in-silico analysis of a dataset of 683 flavonoids for molecular docking to *L. mexicana* pyruvate kinase found that 3-glycosylated flavonoids (seven compounds), 6,8-diglycosyl flavonoids (one compound), and biflavonoids (four compounds) were the most promising ligands [400]. Promastigote surface antigen has been identified as a common protein drug target for *L. braziliensis*, *L. major*, and *L. infantum*. *N*-Acetylglucosamine was identified as a potential lead target molecule based on docking studies (ArgusDock) [401].

**Figure 12:**
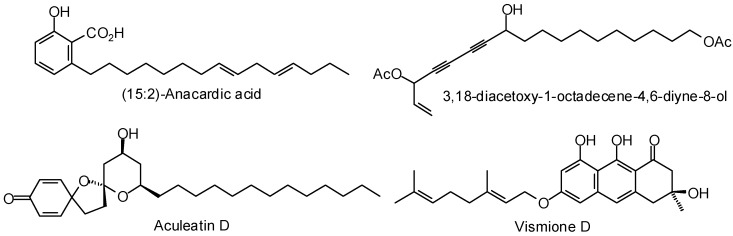
Phytochemical ligands with encouraging docking properties with *Trypanosoma cruzi* silent-information regulator 2 protein 1.

Inhibition of *Trypanosoma cruzi* silent-information regulator 2 proteins (sirtuins) is known to cause arrested growth of the parasite [402]. Sacconnay and co-workers assembled two conformational states of TcSir2rp1 using homology modeling and carried out molecular docking of a library of antitrypanosomal phytochemicals [403]. Four compounds were found to have particularly promising docking characteristics, (15:2)-anacardic acid, 3,18-diacetoxy-1-octadecene-4,6-diyne-8-ol, aculeatin D, and vismione D (Figure 12).

**Figure 13:**
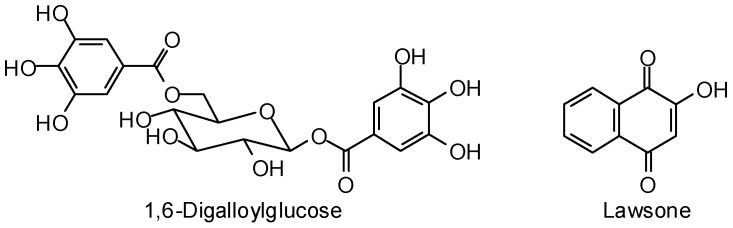
Phytochemical ligands with encouraging docking properties with *Leishmania donovani* adenosine kinase.

*Leishmania* lack the ability to synthesize purines de novo and therefore salvage purines. Adenosine kinase is one of the enzymes in the purine salvage pathway, and *Leishmania* adenosine kinase is crucial for parasite survival [404]. Molecular docking (Glide, FlexX, GOLD) of a library of natural products with a homology-modeled structure of *L. donovani* adenosine kinase has revealed 1,6-digalloylglucose and lawsone (Figure 13) as top hit phytochemical ligands [405].

### 3.2. Plasmodium Targets

Curcumin (Figure 8) has shown antimalarial activity (IC_50_ 5 μM) against *P. falciparum*, and experimental evidence has suggested disruption of parasite microtubules to be responsible for the antiplasmodial activity [406]. Molecular docking studies (AutoDock) have revealed that curcumin interacts with homology-modeled *P. falciparum* tubulin dimer at the colchicine binding site of tubulin rather than the paclitaxel or vinblastine binding sites [406].

The sarco/endoplasmic reticulum Ca^2+^-ATPase orthologue of *P. falciparum* (PfATP6) has been suggested to be a viable drug target for antimalarial chemotherapy [407]. Homology modeling has allowed construction of the three-dimensional structure of PfATP6 and allowed molecular docking/in-silico screening of potential antimalarial drugs, including artemisinin [408] and curcumin [409,410]. Curcumin has also shown selectively strong docking (MolDock) to *L. major* methionyl tRNA synthetase [377]. Bousejra-El Garah and co-workers, however, found no correlation between in silico docking energies to PfATP6 and antimalarial activities of several structurally diverse antimalarial compounds [411].

**Figure 14:**
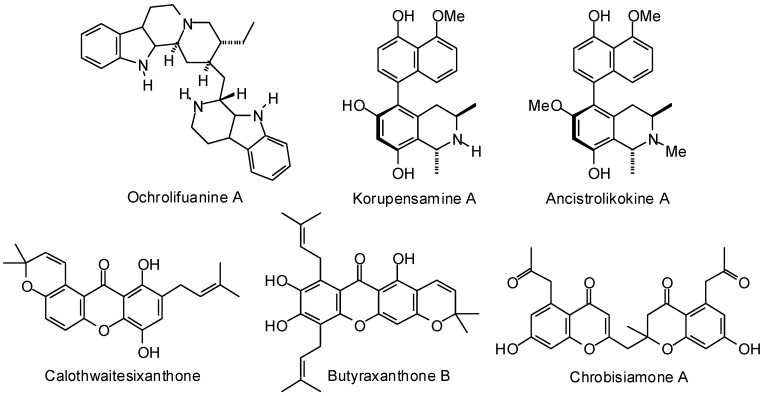

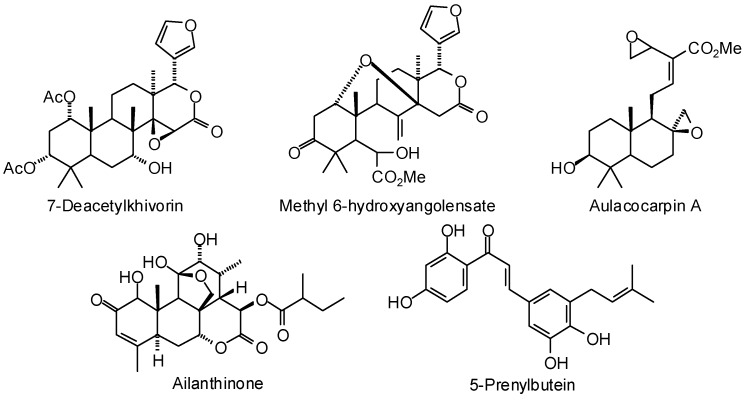
Phytochemical ligands with encouraging docking properties with *Plasmodium falciparum* dihydrofolate reductase.

Kumar and co-workers have carried out a molecular docking examination (AutoDock) of several bioactive natural products with *P. falciparum* dihydrofolate reductase [376]. These workers found ochrolifuanine A, chrobisiamone A, ailanthinone, korupensamine A, butyraxanthone B, ancistrolikokine A, calothwaitesixanthone, 7-deacetylkhivorin, 5-prenylbutein, methyl 6-hydroxy-angolensate, and aulacocarpin A (Figure 14), to show notable docking energies (i.e., lower than the co-crystallized inhibitor WR99210).

**Figure 15:**
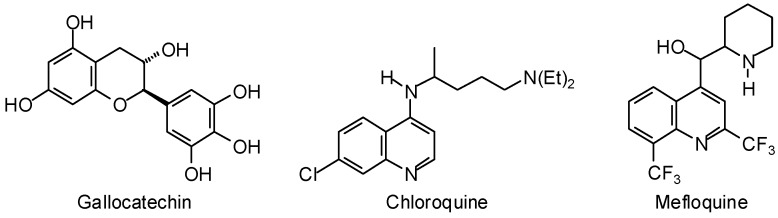
Strongly docking ligands with *Plasmodium falciparum* lactate dehydrogenase.

**Figure 16:**
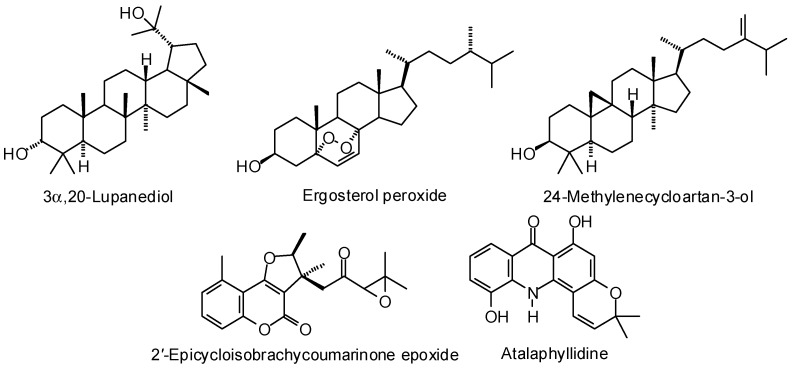
Phytochemical ligands with encouraging docking properties with *Plasmodium* type II fatty acid biosynthesis pathway enzymes.

*Plasmodium* lactate dehydrogenase has been identified as a potential drug target for antimalarials due to parasite dependence on glycolysis for ATP production [412]. Molecular docking of the tea flavonoid gallocatechin (Figure 15) to *P. falciparum* lactate dehydrogenase revealed strong docking, more strongly than either chloroquine or mefloquine, to the NADH binding site of the enzyme [413]. Glycyrrhetic acid (Figure 4) has exhibited notable (IC_50_ 1.69 μg/mL) in vitro antiplasmodial activity against *P. falciparum*, and docking studies (Discovery Studio) have also shown glycyrretic acid to dock moderately well to *P. falciparum* lactate dehydrogenase [414].

(+)-Usnic acid, a secondary metabolite from lichen, was identified as an active and selective inhibitor of the liver stage form of *Plasmodium berghei* by Lauinger and co-workers [415]. In the molecular docking study to identify the binding affinities and binding sites of (+)-usnic acid and three other lichen secondary metabolites (evernic acid, vulpic acid, and psoromic acid, Figure 16) with *Plasmodium* type II fatty acid biosynthesis pathway (FAS-II) enzymes, these workers found that the mechanism of action of lichen acids on FAS-II may be different from those of previously described FAS-II enzymes inhibitors. The modeling study they carried out indicated that those compounds appear to inhibit FAS-II enzymes indirectly via binding to allosteric sites on the protein surface and not to the active sites of FAS-II enzymes. This indirect binding is speculated to possibly affect the enzyme conformations and subsequently interfere with the catalytic activities [415].

Enoyl-acyl carrier protein reductase is a critical enzyme in type II fatty acid biosynthesis in the hepatocyte-stage of *Plasmodium falciparum*. Tallorin and co-workers, based on molecular docking and subsequent in vitro screening, have identified celestrol as a potent PfENR inhibitor [416]. Using molecular docking (AutoDock) coupled with three-dimensional quantitative structure activity relationships (3D-QSAR), Wadhwa and co-workers identified five phytochemicals (3α,20-lupanediol, ergosterol peroxide, 24-methylenecycloartan-3-ol, 2′-epicycloisobrachycoumarinone epoxide, and atalaphyllidine, Figure 16) as potential PfENR inhibitors [417].

**Figure 17:**
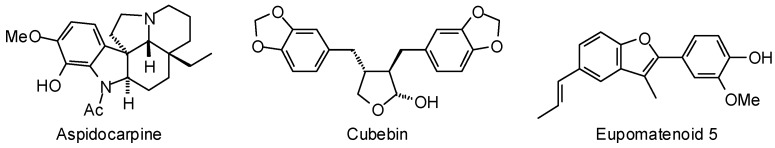
Phytochemical ligands with encouraging docking properties with parasitic mitogen-activated protein kinases.

Recently, Gupta and co-workers have used homology modeling to construct parasitic mitogen-activated protein kinases (MAPKs) for *Leishmania mexicana*, *Plasmodium falciparum*, and *Trypanosoma brucei* [418]. These workers carried out a molecular docking study on a small library of 10 antiparasitic phytochemicals. Of these, aspidocarpine showed excellent docking to both LmxMPK4 and TbMAPK5, and cubebin and eupomatenoid 5 (Figure 17) both docked well with PfMAK2.

## 4. Conclusions

This review has catalogued the numerous druggable parasitic protein targets, with more being identified and three-dimensional structures determined, allowing many potential sites for identification and development of new and selective inhibitors. The theoretical predictions need to be experimentally validated, and the results can be used to guide an effective development of selective and targeted natural products analogues. A perusal of the structures in this review reveals several of the phytochemical ligands with promising docking properties are not likely to have suitable drug-like properties. Therefore, pharmacokinetic and pharmacodymanic studies as well as structure-based design and optimization studies are needed to resolve issues of bioavailability and selectivity. It is advisable to carry out additional filtering for “drug-likeness” [387,419,420], ADME [421], and toxicity prediction [422].

## Figures and Tables

**Table 1:** Protein targets with three-dimensional structures available from the Protein Data Bank (PDB).

Protein Target	PDB Protein Structure						
	*L. donovani*	*L. infantum*	*L. major*	*L. mexicana*	*P. falciparum*	*T. brucei*	*T. cruzi*
Adenine phosphoribosyl transferase (APRT)	1QB7, 1QB8, 1QCC, 1QCD [18]						
Adenosine kinase (AK)						2XTB, 3OTX [19], 4N09 [20]	
Adenoylsuccinate synthetase (AdSS)					1P9B [21]		
Aminopeptidase (Apase)						4EFD; 4FUK [22]	
Apical membrane antigen I (AMA1)					3SRI, 3SRJ, 3ZWZ [23]		
Arginase (ARG)				4ITU, 4IU0, 4IU1, 4IU4, 4IU5 [24]	3MMR [25], 3SL0, 3SL1 [26]		
Arginine kinase (ArgK)							2J1Q [27]
Aspartate aminotransferase (AspAT)					3K7Y [28]		
Autophagy protein 8 (Afg8)					4EOY [29]		
Cathepsin B (CatB)						3HHI [30], 3MOR [31], 4HWY [32]	
Choline kinase (CK)					3F18 [33]		
Cruzain							2AIM [34], 1F29, 1F2A, 1F2B, 1F2C [35], 1ME3, 1ME4 [36], 1U9Q [37], 2OZ2 [38], 3HD3 [39], 3I06 [40], 3IUT [41], 3LXS [42], 4BKL [43], 1EWL; 1EWM; 1EWO; 1AIM [44]
Cyclophilin (Cyp)	2HAQ, 3EOV [45]		2HQJ [46]		1QNG [47]		
Cysteine synthase (CS)			4AIR [48]				
Deoxyuridine triphosphate nucleotidohydrolase (dUTPase)			2CJE, 2YAY, 2YAZ, 2YB0 [49]		1VYQ [50], 2Y8C [51], 3T60, 3T64, 3T6Y, 3T70 [52]	4DK2, 4DK4, 4DKB, 4DL8, 4DLC [53]	1OGK, 1OGL [54]
Diadenosine tetraphosphatase (DATP)						1QJC [55]	
Dihydrofolate reductase-thymidylate synthase (HDFR-TS)					1J3I [56]; 3DGA [57]; 3QGT [58]; 3UM8 [59]; 4DDR, 4DP3, 4DPD, 4DPH [60]	3QFX, 3QGT, 3RG9 [58]	2H2Q, 3CL9, 3CLB [61]; 3HBB [62]; 3KJS [63]
Dihydroorate dehydrogenase (DHODH)	3C61 [64]		3MHU, 3MJY [65]; 3TQ0 [66]; 4EF8, 4EF9 [67]; 3GYE, 3GZ3 [68]		1TV5 [69]; 3I65, 3I68; 3I6R [70]; 3O8A [71]; 3SFK [72]; 4CQ8, 4CQ9, 4CQA [73]	2B4G [74]	3C3N [75]; 2E6D [76]; 2E68, 2E6A, 2E6F, 2DJL, 2DJX [77]; 3W1A , 3W1L, 3W1M, 3W1N, 3W1P, 3W1Q, 3W1R, 3W1T, 3W1U, 3W1X, 3W22, 3W23, 3W2J, 3W2K, 3W2L, 3W2M, 3W2N, 3W2U [78]; 3W3O [79]; 3W6Y, 3W70, 3W71, 3W72, 3W73, 3W74, 3W75, 3W76, 3W7C, 3W7D, 3W7E, 3W7G, 3W7H, 3W7I, 3W7J, 3W7K, 3W7L, 3W7M, 3W7N, 3W7O, 3W7P, 3W7Q, 4JD4, 4JDB [80]; 3W83, 3W84, 3W85 [81]; 3W86, 3W87, 3W88 [82]
d-Tyrosyl-tRNATyr deacylase (DTD)					3KNP, 3KNF, 3KO3, 3KO4, 3KO5, 3KO7, 3KO9, 3KOB, 3KOC [83]; 3LMT, 3LMU, 3LMV [84]; 4NBI, 4NBJ [85]		
Enolase						1OEP [86]; 2PTW, 2PTX, 2PTY, 2PTZ, 2PU0, 2PU1 [87]	
Enoyl acyl-carrier-protein reductase (FabI = ENR)					1NHD, 1NHG, 1NHW, 1NNU, 1VRW [88]; 1UH5, 1V35 [89]; 1ZSN, 1ZW1, 1ZXB, 1ZXL [90]; 2O2S, 2O2Y [91]; 2FOI, 2NQ8, 2OL4, 2OOS, 2OP0, 2OP1 [92]; 3LSY, 3LT0, 3LT1, 3LT2, 3LT4 [93]; 4IGE, 4IGF [94]		
Falcipain 2 (FP-2)					1YVB [95]; 2GHU [96]; 2OUL [97]; 3BPF [98]; 3PNR [99]		
Falcipain 3 (FP-3)					3BPM [98]; 3BWK [38]		
Farnesyl diphosphate synthase (FPPS)			4K10, 4JZX, 4JZB [100]			2EWG, 2I19 [101]; 2P1C [102]; 3DYF, 3DYG, 3DYH, 3EFQ, 3EGT [103]; 2OGD [104]	1YHL, 1YHM [105]; 3IBA, 3ICK, 3ICM, 3ICN, 3ICZ, 3ID0 [106]; 4DWB, 4DWG, 4DXJ, 4DZW, 4E1E [107]
Ferredoxin-NADP+ reductase (FNR)					2OK7, 2OK8 [108]		
FK506 binding protein (FKBP35)					4J4N [109]		
Fructose-1,6-bisphosphate aldolase (ALDO)				1EPX [110]; 2QAP, 2QDG, 2QDH [111]	1A5C [112]		
Glutamate dehydrogenase 2 (GDH2)					3R3J [113]		
Glutathione peroxidase-like enzyme 1 (GPX1)							3E0U [114]
Glutathione reductase (GR)					1ONF [115]		
Glutathione *S*-transferase (GST)					1OKT [116]; 1PA3, 1Q4J [117]; 2AAW [118]		
Glyceraldehyde 3-phosphate dehydrogenase (GAPDH)				1GYP [119]; 1A7K [120]; 1GYQ [121]	1YWG [122]; 2B4R, 2B4T [123]	2X0N [124]; 4P8R [125]	1K3T [126]; 1ML3 [127]; 1QXS [128]; 3IDS [129]
Glycerol-3-phosphate dehydrogenase (GPDH)				1EVY, 1EVZ [130];1JDJ, 1M66, 1M67, 1N1G [131]; 1N1E [132]			
Glyoxalase I (GLO1)			2C21 [133]				
Glyoxalase II (GLO2)		2P18, 2P1E [134]					
GMP synthetase (GMPS)					3UOW [135]		
Guanylate kinase (GK)					1Z6G [136]		
Heat shock protein 90 (HSP90)			3H80 [137], 3Q5J, 3Q5K, 3Q5L, [138]; 3U67 [139]				
Histidyl-tRNA synthetase (HisRS)						3HRI [140]	3HRK, 3LC0 [140]
Histo-aspartic protease (HAP)					3FNS, 3FNT, 3FNU [141]		
β-Hydroxyacyl-acyl carrier protein Dehydratase (FabZ)					3AZ8, 3AZ9, 3AZA, 3AZB [142]		
(*E*)-4-hydroxy-3-methyl-but-2-enyl-diphosphate reductase (LytB)					4N7B [143]		
Hypoxanthine-guanine phosphoribosyl transferase (HGPRT)					1CJB [144]		1TC1, 1TC2 [145]; 1P19 [146]
Lactate dehydrogenase (LDH)					1LDG [147]; 1CEQ, 1CET [148]; 1T24, 1T25, 1T26, 1T2C, 1T2D [149]; 1U4O, 1U4S, 1U5A, 1U5C, 1XIV [150]; 2A94 [151]; 4B7U [152]		
Lipoamide dehydrogenase (LADH)							2QAE [153]
Lysyl-tRNA synthetase (Lys-RS)					4H02 [154]		
M1 amino peptidase (A-M1)					4K5L, 4K5M, 4K5N, 4K5O, 4K5P [155]		
M17 amino peptidase (A-M17)					4K3N [155]; 3KQX, 3KQZ, 3KR4, 3KR5 [156]; 3T8V, 3T8W [157]		
M18 aspartyl aminopeptidase (M18AAP)					4EME [158]		
Macrophage infectivity potentiator (MIP)							1JVW [159]
Metacaspase-2 (MCA2)						4AF8, 4AFP, 4AFV [160]	
Metallocarboxypeptidase 1 (MCP-1)							3DWC [161]
Methionine aminopeptidase 1b (MAP1b)					3S6B [162]		
Methionyl-tRNA synthetase (MetRS)			3KFL [163]			4EG1, 4EG3, 4EG4, 4EG5, 4EG6, 4EG7, 4EG8, 4EGA [164]; 4MVW, 2MVX, 4MVY, 4MW0, 2MW1, 4MW2, 4MW4, 4MW5, 4MW6, 4MW7, 4MW9, 4MWB, 4MWC, 4MWD, 4MWE [165]	
Mitogen-activated protein kinase (MAPK)			3PGI, 3UIB [166]				
*N*^5^,*N*^10^-Methylenetetrahydrofolate dehydrogenase/cyclohydrolase (DHCH)			4A26 [167]				
Nicotinamidase (PnC1)		3R2J [168]					
*N*-Myristoyl transferase (NMT)	2WUU [169]		3H5Z, 2WSA [170]; 4A2Z, 4A30, 4A31, 4A32, 4A33 [171]				
Nucleoside 2-deoxyribosyltransferase (NDRT)						2A0K, 2F2T, 2F62, 2F64, 2F67 [172]	
Nucleoside diphosphate kinase B (NDKB)			3NGR, 3NGS, 3NGT, 3NGU [173]			4FKX, 4FKY [174]; 4F4A, 4F36 [175]	3NGR, 3NGS, NGT, 3NGU, 3PRV [173]
Nucleoside hydrolase (NH)			1EZR [176]				
Inosine-Adenosine-Guanosine nucleoside hydrolase (IAGNH)						4I70, 4I71, 4I72, 4I73, 4I74, 4I75 [177]	
Inosine-Guanosine nucleoside hydrolase (IG-NH)						3FZ0, 4I70, 4I71, 4I72, 4I73, 4I74, 4I75 [178]	
Nucleosome assembly protein (NapL)					3FS3 [179]; 3GYV, 3GYW [180]		
Old yellow enzyme (OYE)							3ATY, 3ATZ [181]; 4E2B, 4E2D [182]
Oligopeptidase B (OPB)			2XE4 [183]			4BP8, 4BP9 [184]	
Ornithine decarboxylase (ODC)						1QU4 [185]; 1F3T [186]; 1NJJ [187]	
Ornithine δ-aminotransferase (OAT)					3NTJ, 3LG0 [188]		
Orotidine 5′-monophophate decarboxylase (OMPDC)		3QW3 [189]			2QAF, 2Q8Z, 3BAR [190]; 2ZCG [191]; 2ZA1, 2ZA2, 2ZA3 [192]; 3S9Y [193]; 3VI2 [194]; 2Q8L [195]; 2F84 [196]; 3MWA, 3N2M, 3N34, 3N3M [197];		
Oxoacyl acyl-carrier-protein reductase (OAR)					2C07 [198]		
Peptide deformylase (PDF)					1JYM [199]; 1RL4, 1RQC [200]		
Peroxisomal targeting signal 1 (PTS1)						3CV0, 3CVL, CVN, 3CVP, 3CVQ [201]	
Peroxisomal targeting signal 2 (PTS2)						2F2J [110]	
Phosphethanolamine methyltransferase (PMT)					3UJ6, 3UJ7, 3UJ8, 3UJ9, 3UJA, 3UJB [202]		
Phosphodiesterase B1 (PDEB1)			2R8Q [203]			4I15 [204]	
Phosphodiesterase C (PDEC)							3V93 [205]; 3V94 [206]
Phosphoenolpyruvate carboxykinase (PEPCH)							1II2 [207]
Phosphofructokinase (PFK)						3F5M [208]	
6-Phosphoglucolactonase (6PGL)						2J0E [209]; 3E7F, 3EB9 [210]	
6-Phosphogluconate dehydrogenase (6PGDH)						1PGJ [211]	
Phosphoglucose isomerase (PGI)				1Q50, 1T10 [212]		2O2C, 2O2D [213]	
Phosphoglycerate kinase (PGK)					3OZA, 3OZ7 [214]	13PK [215]; 16PK [216]	
Phosphoglycerate mutase (PGAM)				3IGY, 3IGZ [217]	3EOZ [218]	3NVL [219]	
Phosphomannomutase (PMM)				2I54, 2I55 [220]		3F9R [221]	
Plasmepsin I (PMI)					2R9B [222]; 3QRV, 3QS1 [223];		
Plasmepsin II (PMII)					1SME [224]; 1LEE, 1LF2 [225]; 1LF3, 1LF4 [226]; 2BJU [227]; 2IGX, 2IGY [228]; 3F9Q [229]; 1M43 [230]; 1ME6 [231]; 1W6H, 1W6I [232]; 1XDH, 1XE5, 1XE6 [233]		
Plasmepsin IV (PMIV)					1LS5 [225]		
Proline racemase (PRACA)							1W61, 1W62 [234]
Protein Kinase 5 (PK5)					1OB3, 1V0O, 1V0P [235]		
Protein tyrosine phosphatase 1 (PTP1)						3M4U [236]	4AZ1 [237]
Pteridine reductase 1 (PTR1)	2XOX [238]		1E7W, 1E92 [239]; 1W0C [240]; 2BF7, 2BFA,2BFM, 2BFO, 2BFP [241]; 2QHX, 3H4V [242]			2C7V [243]; 2WD7, 2WD8, 3GN1, 3GN2 [244]; 2VZ0 [245]; 3BMC, 3BMN, 3BMO, 3BMQ, 3JQ6, 3JQ7, 3JQ8, 3JQ9, 3JQA, 3JQB, 3JQC, 3JQD, 3JQE, 3JQF, 3JQG [246]; 2X9N, 2X9G, 2X9V, 3MCV [247]; 2YHI [248]	
Pteridine reductase 2 (PTR2)							1MXF, 1MXH [249]
Purine nucleoside phosphorylase (PNP)					1NW4, 1Q1G [250]; 2BSX, 1SQ6 [251]; 3ENZ [252]		
Pyridoxal kinase (PdxK)						3ZS7 [253]	
Pyruvate kinase (PYK)				1PKL [254]; 3E0V, 3E0W [255]; 3IS4, 3KTX [256]; 3HQN, 3HQO, 3HQP, 3HQQ [257]; 3PP7, 3QV6, 3QV7, 3QV8, 3QV9 [258]; 3SRK [259]	3KHD [260]	4HYV, 4HYW [261]; 4KCT, 4KCU, 4KCV, 4KCW [262]	3PP7, 3QV6, 3QV7, 3QV8, 3QV9 [258]
Rhodesain						2P7U [38], 2P86 [263]	
Ribose 5-phosphate isomerase type B (RPIB)							3K7O, 3K7S, 3K8C, 3M1P [264]
Ribulose 5-phosphate 3-epimerase (a1RPE)					1TQX [265]		
RNA Editing ligase 1 (REL1)						1XDN [266]	
*S*-Adenosylhomocysteine hydrolase (SAHH)			3G1U [267]		1V8B [268]	3H9U [269]	
Seryl-tRNA synthetase (SerRS)						3LSQ, 3LSS [270]	
Sirtuin 2A (Sir2A)					3U31, 3U3D [271]		
Spermidine synthase (SpdSyn)					2HTE [136] ; 2I7C, 2PSS, 2PT6, 2PT9 [272]; 3B7P; 2PWP [273]; 3RIE [274]		3BWC [275]
Sterol 14-α Demethylase (CYP51)		3L4D [276]				3G1Q, 3GW9 [277]; 2WV2, 2X2N [278]; 3P99 [279]; 3TIK [280]; 4BJK [281]; 4G7G, 4G3J [282];	2WUZ, 2WX2 [278]; 3K1O, 3KHM, 3KSW [283]; 4H6O [284]; 3ZG2, 3ZG3 [285]; 4COH [286]; 4BY0 [287] ; 4BMM [288]
Sterol carrier protein, type 2 thiolase (SCP2-thiolase)				3ZBG, 4B19 [289]		4BI9 [289]	
Superoxide dismutase (SOD)					2BPI [290]	3ESF [291]	2GPC [291]
Terminal RNA uridyltransferase (TUTase)						2B4V, 2B51, 2B56 [292]; 2IKF, 2NOM [293]; 2Q0C, 2Q0D, 2Q0E, 2Q0F, 2Q0G [294]	
Thiamine phosphate synthase (TPS)						2Y6Z [295]	
Thiol-dependent reductase 1 (TDR1)		4AGS [296]					
Thioredoxin reductase (TrxR)					4J56, 4J57 [297]		
Thymidylate kinase (TMPK)					2WWF, 2WWG, 2WWH, 2WWI [298]; 2YOF, 2YOG, 2YOH [299]		
Transkelolase (Tk)				1R9J [300]			
Translationally controlled tumor protein (TCTP)					3P3K [301]		
*trans*-Sialidase (TS)							1MS0, 1MS1, 1MS3, 1MS4, 1MS5, 1MS8, 1MS9, 1MR5 [302]; 1S0I, 1S0J, 2AH2 [303]; 3B69 [304]; 3OPZ [305]
Triosephosphate isomerase (TIM)				1AMK [306]; 1IF2; [307]; 1N55 [308]; 2VXN [309]; 2Y61, 2Y62, 2Y63 [310]	1YDV [311]; 1LYX, 1LZO [312]; 1M7O, 1M7P [313]; 1O5X [314]; 2VFI [315]	1AG1 [316]; 3TIM [317]; 1IIG, 1IIH; 6TIM [318]; 5TIM [319]; 4TIM [320]; 1TPD, 1TRD, 2V5L [321]; 1TPE, 1TPF [322]; 1ML1 [323]; 1DKW [324]; 2J24, 2J27 [325]; 2X1U [326]	1TCD [327]; 1CI1 [328]; 1SUX [329]; 2OMA [330]; 2V5B [331]; 3Q37 [332]; 4JEQ [333]
Trypanothione reductase (TR)		2JK6, 2W0H [334]; 2X50 [335]; 2YAU [336]; 4ADW, 4APN [337]				2WBA [338]; 2WOI, 2WOV, 2WOW, 2WP5, 2WP6, 2 WPC, 2WPE, 2 WPF [339]; 4NEV [340];	1NDA [341]; 1AOG [342]; 1BZL [343]; 1GXF [344]; 4NEW [340]
Tryparedoxin-dependent peroxidase (TDPX)						2VUP [345]	4LLR [346]
Tryptophanyl-tRNA synthetase (TrpRS)					4J75, 4J76 [347]	3I05 [348]	
Tyrosyl-tRNA synthase (TyrRS)			3P0H, 3P0I, 3P0J [349]		3VGJ [350]		
Ubiquitin and Nedd8 Hydrolase (UCHL3)					2WDT, 2WE6 [351]		
UDP-Galactose 4′-epimerase (UGE)						1GY8 [352]	
UDP-Galactopyranose mutase (UGM)							4DSG, 4DSH [353]
UDP-glucose pyrophosphorylase (UGP)			4M28, 4M2A, 2OEF, 2OEG [354]				
UDP-N-acetylglucosamine pyrophosphorylase (UAP)						4BQH [355]	
UMP synthase (UMPS)	3QW4 [189]						
Uridine phophorylase (UP)						3BJE [356]	

**Table 2:** Popular molecular docking programs used for virtual screening of natural product libraries.

Docking Program	Source
AutoDock	Scripps Research Institute, http://autodock.scripps.edu/ [369]
Molegro Virtual Docker	Molegr ApS (no longer available) [370]
GLIDE	Schrödinger, https://www.schrodinger.com/Glide/ [371]
AutoDock Vina	Scripps Research Institute, http://vina.scripps.edu/ [372]
Molecular Operating Environment (MOE)	Chemical Computing Group, http://www.chemcomp.com/MOE-Molecular_Operating_Environment.htm
CDOCKER (Discovery Studio)	Dassault Systèmes BIOVIA, http://accelrys.com/products/collaborative-science/biovia-discovery-studio/
ArgusLab	http://www.arguslab.com/arguslab.com/ArgusLab.html
iGemDock	National Chiao Tung University, http://gemdock.life.nctu.edu.tw/dock/download.php
Surflex-Dock	Certara USA, Inc., https://www.certara.com/ [373]
GOLD	Cambridge Crystallographic Data Centre (CCDC), http://www.ccdc.cam.ac.uk/solutions/csd-discovery/components/gold/
FlexX	BioSolveIT, http://www.biosolveit.de/FlexX/
